# Who should be vaccinated first? Comparing vaccine prioritization strategies in Israel and European countries using the Covid-19 Health System Response Monitor

**DOI:** 10.1186/s13584-021-00453-1

**Published:** 2021-02-19

**Authors:** Jonathan Cylus, Dimitra Panteli, Ewout van Ginneken

**Affiliations:** 1 European Observatory on Health Systems and Policies, London Hub, London School of Economics and London School of Hygiene and Tropical Medicine, London, UK; 2 European Observatory on Health Systems and Policies, Brussels, Belgium; 3 European Observatory on Health Systems and Policies, Berlin hub, Berlin University of Technology, Berlin, Germany

**Keywords:** (3–10), Covid-19, Israel, vaccination, age, priority-setting.

## Abstract

The rapid rollout of Israel’s vaccination program has led to considerable international interest. In this brief commentary we consider how the criteria for vaccination priority groups differ between Israel and selected European countries. We argue that following the Israeli approach of using broad criteria for prioritization— i.e. having fewer groups and a lower age threshold— could have several beneficial effects, including more manageable logistics and fewer roll out delays, as well as potentially reducing pressure on hospitals. With an increasing supply of vaccines becoming available rapidly in much of Europe, countries could consider following the approach of Israel and adopting broader priority criteria going forward.

Israel has been lauded internationally for its vaccine rollout, administering a first dose to over 62% of its population as of 8 February while the majority of EU countries are between 3 and 5% [1]. While it still faces challenges including high infection rates and vaccine hesitancy, there are many factors described in the paper by Rosen et al. that have contributed to Israel’s rapid rollout [2]. Some of these would be challenging if not impossible for many European countries to replicate, especially at this stage of the pandemic response. These include the country’s small size, high population density, a relatively young population, Israel’s willingness to share data with manufacturers in return for early vaccine access and being accustomed to operating on an emergency preparedness footing (due to security risks). Most importantly, the EU countries, which decided to jointly purchase vaccines to prevent an ugly competition for doses, cannot realistically replicate Israel’s speed in procuring enough doses of vaccine to cover their combined population of 446 million people given how tight the global market is. Yet the relatively large differences in the proportions of people who had their first dose administered, which ranges from less than 2% in Latvia and Bulgaria to over 5% in Malta and Denmark [1], means that some countries have been more effective than others at rolling out their vaccination programs; some of this variation could reflect country-specific prioritization strategies.

McKee & Rajan [3] have highlighted several characteristics of the Israeli vaccine rollout which countries in the European region may want to consider in their own vaccination strategies, including responsibilities for the delivery of vaccination services and the availability of corresponding workforce. Here we consider how selected country strategies in Europe have compared to Israel’s vaccine rollout strategy, focusing on the prioritization of population groups for vaccination. We draw on Rosen et al. and on information collected for European countries in the Covid-19 Health System Response Monitor (HSRM) [4].

## The eligibility criterion used for the first phase of the rollout varies across countries

Arguably one of the reasons for the rapid vaccine rollout in Israel is that it has devised very simple vaccination priority criteria that simultaneously target individuals at highest risk of death, at highest risk of hospitalization, and those in most frequent contact with cases. Rosen et al. report that Israel defined four broad vaccination groups for its rollout: those age 60 and over; those with certain pre-existing conditions; nursing home residents; and front-line health workers. This simple but far-reaching strategy contrasts with the approaches of some European countries.

One of the most notable rollout strategy differences is that some European countries have first focused their attention on frontline health workers and/or care home residents before the general population above a pre-defined age, in line with WHO guidance developed for a context with limited vaccine supply [5]. This approach recognizes not only that these groups are at a high risk of exposure to Covid-19, but also that in an overstretched health workforce every professional is needed, especially in ICUs.

For example, in Slovakia health workers, medical students, social service home staff, armed forces and some infrastructure workers are being offered the vaccine in the first round, while patients over age 65 and the chronically ill are not eligible until the second round. Spain is also prioritizing residents and workers in assisted-living nursing homes, as well as care centres for highly-disabled people in addition to front line healthcare and social care workers. Only after vaccination of these groups is completed, other health and social care workers and non-institutionalised highly dependent people will be offered the vaccine.

In Latvia health workers treating COVID-19 patients and professionals working in Emergency Medical Services (EMS) are in the top priority group. Lithuania also prioritizes those who work directly with Covid-19 patients. The Dutch Health Council originally recommended a strategy comparable to the Israeli rollout. However, following lobbying efforts from the hospital sector, which used the delayed roll out and the prospect of a third wave to its political advantage, this was changed to first prioritize hospital and nursing home personnel over other groups, including older people and those with pre-existing conditions. Therefore, one key difference that we note is that in contrast to Israel, some European countries have not decided to prioritize people for vaccination based on age.

## Among those who do consider age as a priority criterion, some set high initial age thresholds

There are also differences across countries in the age threshold used for vaccine prioritization. Among those countries that prioritize people based on age, many countries have focused their attention first on the oldest old, in contrast to Israel’s initial age cut-off of 60 years and over. Austria, Germany and the United Kingdom prioritize those 80 years and over before planning to move incrementally to younger age groups. Germany prioritizes those 70 years and over in its second phase and those 60 years and over in its third phase. Estonia focuses first on those 70 years and over. Portugal in its first phase is including people age 50 years or older, but only if they have one of a short list of chronic conditions. Sweden, which initially had an age criterion simpler than the Israeli one, set the age threshold to be all adults over 18 being vaccinated in the first half of 2021, reversed course on 4 February and now prioritizes those above 65.

While there is no definitive rationale for various countries’ prioritization strategies, it should be noted that although the mortality risk is highest at very old ages and among care home residents, ICUs in many countries are currently treating relatively younger patients. In the Netherlands, for example, the three most represented age groups (5-year cohorts) in ICUs are between age 60 and 75 [6] This suggests that any approach that ignores these age groups may do little to alleviate pressures on health systems, especially if countries decide to re-open prematurely and infections among younger groups rise.

## An increasing supply of vaccines allows for broader criteria going forward

Naturally many countries have designed their vaccine rollout strategy based on their own expectations about their vaccine supply. Scarcity concerns have resulted in narrow criteria in this early phase of the rollout. Additionally, although it is not decisively demonstrated that those who are vaccinated stop transmitting Covid-19, in the event that some degree of transmission is reduced through vaccination, it could make sense from a societal perspective to focus on narrowly defined groups who also are most likely to contribute to community spread.

This being said, there are valuable takeaways from the simplicity of Israel’s approach to logistics, especially regarding the second quarter of 2021, when the scarcity of the vaccine is expected to become less of a problem in the EU. A simple set of eligibility criteria, with an age cut off that is not limited to the oldest old, can be beneficial for a number of reasons. These range from more manageable logistics needed to determine eligibility to the likelihood of people being aware of when they are eligible to be vaccinated. The latter is particularly important for countries that require people to register for vaccination services, such as Hungary and Greece. It could also help prevent discord among the population caused by choices in prioritizations. Furthermore, a broad strategy may have a more immediate impact on alleviating pressures on hospitals and ICU units. Lastly, if countries are resigned to offering the vaccine only to narrowly defined groups before broadening the criteria, they may have to contend with significant delays in rollout as they face diminishing returns trying to reach all eligible individuals before proceeding to the next phase.

The pandemic has by necessity created a number of natural experiments, with countries trying to design the best possible vaccine rollout based on several determinants, like vaccine supply and a range of country characteristics, as Rosen et al. clearly describe for Israel. Ongoing comparative research will hopefully provide more insights into which approach is most effective so we can be better prepared in the event of a future pandemic.
